# The Role of Socioeconomic Status in Influencing Stage at Presentation and Overall Survival in Bladder Cancer: Experience From the West of Scotland

**DOI:** 10.7759/cureus.103399

**Published:** 2026-02-11

**Authors:** Jamie Leask, Ibrahim Ibrahim, Blair Wilson, Jane Hendry, Abdullah Zreik

**Affiliations:** 1 Urology, Queen Elizabeth University Hospital, Glasgow, GBR

**Keywords:** bladder cancer, different socio-economic groups, overall survival (os), retrospective cohort, socio-economic impact

## Abstract

Introduction

Bladder cancer is responsible for significant reductions in health-related quality of life and societal and economic burden. Despite this, large-scale studies on the relationship between socioeconomic differences and bladder cancer outcomes are lacking. In light of this, we conducted a regional retrospective cohort analysis to determine whether deprivation had an impact on the stage at presentation or overall survival (OS) in patients with bladder cancer.

Methods

Demographic and clinical information were collated on every patient diagnosed with bladder cancer (all TNM stages) in the West of Scotland over five years (n = 3,089). The Scottish Index of Multiple Deprivation (SIMD) quintiles were used as a surrogate for socioeconomic status. Multivariable logistic regression and Cox models were applied to evaluate the impact of social deprivation on both the stage at presentation and OS in bladder cancer patients.

Results

In the multivariate Cox model, the less deprived had a lower hazard of death (hazard ratio (HR): 0.80, 95% confidence interval (CI): 0.70-0.91, p < 0.05). The proportion presenting with muscle invasive bladder cancer MIBC was 24.6% in the more deprived group (SIMD: 1-3; 512/2,078), and 24.4% in the less deprived group (SIMD: 4-5; 247/1,011), p = 0.94. More females presented with MIBC (28.3%; 252/890) than males (23.1%; 507/2,199). Muscle-invasive disease was strongly associated with mortality (HR: 4.97, 95% CI: 4.38-5.62, p < 0.05). Age was independently associated with mortality (HR: 1.04 per year, 95% CI: 1.04-1.05, p < 0.05). Sex was not associated with OS after adjustment (HR: 0.97, 95% CI: 0.85-1.11, p = 0.70).

Conclusions

Lower socioeconomic status was associated with a negative effect on overall survival in bladder cancer within this cohort, conferring an approximately 25% higher hazard of death. These findings are consistent with previous studies. Female patients were found to present with more advanced disease, which also correlates with existing literature. However, to the best of our knowledge, this is the first study to demonstrate that socioeconomic status in patients with bladder cancer does not influence stage at presentation. This finding contradicts existing evidence and warrants further investigation.

## Introduction

Health inequalities are systemic across populations, with socioeconomic status being a principal driver of such discrepancies. Despite an increase in overall life expectancy over the past several decades, the social divide between the top and bottom persists in higher-income countries [1]. Multiple public health reports [2-4] have supported the concept of the inverse care law, whereby those who require the most care often receive the least amount of care [5]. Outcomes in cancer care and survivorship are no exception [6].

Bladder cancer is the 11th most common cancer in the UK and the ninth most common cancer globally, representing a significant societal and economic burden. It is estimated that the treatment of bladder cancer accounts for approximately 5% of total healthcare cancer expenditure in the European Union [7]. In contrast, in the United States, it is the most expensive cancer to treat on a per-patient basis [8]. There are striking differences in outcomes between muscle-invasive (MIBC) and non-muscle-invasive (NMIBC) bladder cancer. The latter is characterised by a good prognosis despite a high recurrence rate, while the former is associated with significantly lower survival rates [9]. As with many other malignancies, there is a male preponderance. However, bladder cancer is the only common malignancy in which females have worse cancer-specific outcomes than men. There is some evidence to suggest that this discrepancy is confined to females from more deprived areas [10].

Developed countries are increasingly applying standardised census-based deprivation indices as a means of quantifying health outcomes [9,11,12]. Such multiple deprivation indices provide a more multifaceted perspective than could be gained by a single variable, such as income or educational attainment [13]. The Scottish Index of Multiple Deprivation (SIMD) is an area-based measure of relative deprivation. It stratifies areas into quintiles (1 = most socially deprived, to 5 = least socially deprived), by evaluating seven domains: income, employment, education, health, access to services, crime, and housing [14]. In this study, we aimed to evaluate whether social deprivation as dictated by the SIMD had an impact on stage at presentation and overall survival (OS) in a large cohort of consecutive patients diagnosed with bladder cancer in the West of Scotland.

## Materials and methods

We conducted a retrospective cohort study of consecutive patients diagnosed with bladder cancer in the West of Scotland region between April 2018 and June 2023. Any patient diagnosed with bladder cancer (any stage or grade) was included in the study. Patients without the relevant clinical and demographic information were excluded from the study. Of 3,121 patients with a transurethral resection of bladder tumour (TURBT) data, 3,089 had complete data for deprivation banding, tumour stage, age, and sex, and so were included in adjusted analyses. Relevant clinical and demographic information was obtained from patient records. 

Socioeconomic deprivation was measured using the SIMD quintiles, which were divided into two groups: more deprived (SIMD 1-3) and less deprived (SIMD 4-5). Pathological tumour stage strings were separated into non-muscle invasive (Tis, Ta, T1) and muscle-invasive (T2-4) bladder cancer (NMIBC vs. MIBC). The primary outcome was OS from the date of index TURBT to death from any cause. The secondary outcome was stage at presentation (NMIBC vs. MIBC).

Statistical analysis

We summarised cohort characteristics descriptively. The association between deprivation and stage at presentation was assessed using a Chi-square test of independence on SIMD group (1-3 vs. 4-5) x T group (NMIBC vs. MIBC). Adjusted OS was visualised with Kaplan-Meier curves by SIMD group. Cox proportional hazards regression estimated adjusted hazard ratios (HRs) and 95% confidence intervals (CIs) for less deprived vs. more deprived, T group (NMIBC vs. MIBC), age, and sex. A two-sided alpha level of 0.05 was used throughout. For reporting, p < 0.05 is shown for all p-values <0.05; otherwise, p-values are rounded to two decimal points.

Primary analyses were performed programmatically in Python v3.9 (Centrum Wiskunde & Informatica, Amsterdam, Netherlands) using statsmodels (Cox proportional hazards with Efron ties), SciPy (Chi-square testing), and matplotlib (Kaplan-Meier plots). For transparency and reproducibility, equivalent IBM SPSS Statistics Syntax (IBM Corp., Armonk, NY) is provided to replicate the crosstabs, Kaplan-Meier curves, and adjusted Cox model. Results are consistent across implementations.

## Results

A total of 3,089 patients were included in adjusted analyses. Baseline demographics across the two SIMD groupings, in addition to the SIMD quintile distributions of the cohort, are shown in Table 1 and Table 2, respectively. There were 1,021 deaths by 2024-05-29. Median follow-up duration among censored patients was 1,223 days.

**Table 1:** Baseline demographics of cohort across more and less deprived SIMD groups SIMD: Scottish Index of Multiple Deprivation; SD: standard deviation; IQR: interquartile range

Characteristic	Overall	More deprived (SIMD 1-3)	Less deprived (SIMD 4-5)
N	3,089	2,080	1,009
Age: mean (SD)	71.6 (10.8)	71.1 (10.9)	72.8 (10.5)
Age: median (IQR)	73.0 (65.0 – 80.0)	72.0 (64.0 – 79.0)	74.0 (67.0 – 80.0)
Male: n (%)	2202 (71.2%)	1461 (70.2%)	741 (73.3%)
Female: n (%)	890 (28.8%)	620 (29.8%)	270 (26.7%)

**Table 2:** SIMD quintile distribution of the cohort SIMD: Scottish Index of Multiple Deprivation

SIMD quintile	N	%
1	893	28.9
2	669	21.6
3	520	16.8
4	501	16.2
5	506	16.5

Deprivation and stage at presentation

As demonstrated in Table 3, the proportion presenting with MIBC was 24.6% in the more deprived group (SIMD 1-3; 512/2,078), and 24.4% in the less deprived group (SIMD 4-5; 247/1,011), p = 0.94. Thus, deprivation was not associated with increased odds of MIBC at diagnosis. As shown in Table 4, more females presented with MIBC (28.3%; 252/890) than males (23.1%; 507/2,199). 

**Table 3:** SIMD group vs. stage at presentation SIMD: Scottish Index of Multiple Deprivation; NMIBC: non-muscle invasive bladder cancer; MIBC: muscle invasive bladder cancer

SIMD grouping	NMIBC	MIBC
SIMD 1-3 (more deprived)	1,568	512
SIMD 4-5 (less deprived)	762	247

**Table 4:** Sex vs. stage at presentation (NMIBC vs. MIBC) NMIBC: non-muscle invasive bladder cancer; MIBC: muscle invasive bladder cancer

Sex	NMIBC	MIBC
Female (n)	638	252
Male (n)	1,692	507

Overall survival (OS)

**Figure 1:**
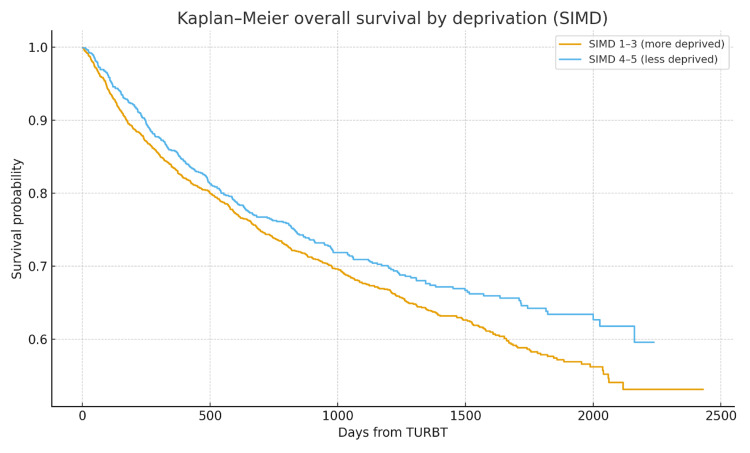
Kaplan-Meier OS by SIMD deprivation indices (1-3 vs. 4-5) HR: 0.80, 95% CI: 0.70-0.91, p < 0.05 OS: overall survival; SIMD: Scottish Index of Multiple Deprivation; TURBT: transurethral resection of bladder tumour; HR: hazard ratio; CI: confidence interval

**Figure 2:**
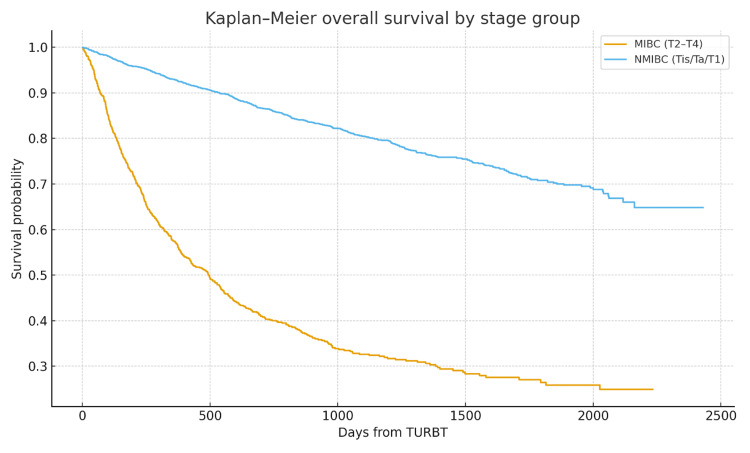
Kaplan-Meier OS based on NMIBC vs. MIBC HR: 4.97, 95% CI: 4.38-5.62, p < 0.05 OS: overall survival; NMIBC: non-muscle invasive bladder cancer; MIBC: muscle invasive bladder cancer; TURBT: transurethral resection of bladder tumour; HR: hazard ratio; CI: confidence interval

As demonstrated in Table 5, in the multivariate Cox model, the less deprived had a lower hazard of death (HR: 0.80, 95% CI: 0.70-0.91, p < 0.05). This indicates significantly worse OS in the more deprived group, with a ~25% higher hazard (Figure 1). MIBC was strongly associated with mortality (HR: 4.97, 95% CI: 4.38-5.62, p < 0.05) as shown in Figure 2. Age was independently associated with mortality (HR: 1.04 per year, 95% CI: 1.04-1.05, p < 0.05). Sex was not associated with OS after adjustment (HR: 0.97, 95% CI: 0.85-1.11, p = 0.70).

**Table 5:** Cox proportional hazards model adjusted for age, deprivation group, stage at presentation and sex HR: hazard ratio; CI: confidence interval; NMIBC: non-muscle invasive bladder cancer; MIBC: muscle invasive bladder cancer

Variable	HR	95% CI	P-value
Age (per year)	1.04	1.04 - 1.05	< 0.05
Less vs. more deprived	0.80	0.70 - 0.91	< 0.05
NMIBC vs. MIBC	4.97	4.38 - 5.62	< 0.05
Male vs. female	0.97	0.85 - 1.11	0.70

## Discussion

Our analysis highlighted several key findings. Social deprivation did not shift stage at presentation towards muscle-invasive disease in this cohort, with the proportions of NMIBC vs. MIBC being essentially identical between more and less deprived groups. Approximately 25% of patients with bladder cancer present with muscle-invasive disease [9], which reflects the figures in this cohort study. It has also long been recognised that females present with more advanced disease [15]. Again, this is in line with our figures. Deprivation was associated with poorer OS, with patients in SIMD 1-3 experiencing approximately 25% higher adjusted hazard of death compared to SIMD 4-5. Unsurprisingly, stage at presentation was the most dominant prognostic factor, with MIBC conferring approximately five-fold higher mortality than NMIBC. Increasing age was also independently associated with worse OS.

A United States population-based analysis evaluated the impact of the Area Deprivation Index (ADI) in patients with MIBC only [11]. In a cohort of 2,597 patients, they reported worse OS in patients from deprived areas but not worse cancer-specific survival (CSS). This contrasted with previous studies [16,17] where both OS and CSS were inferior in socioeconomically deprived populations. However, this was felt to relate to increased migration of MIBC to centres of excellence. It has been shown that treatment in this context does not confer differences in CSS irrespective of socioeconomic status [18]. Further comparison to our data is somewhat limited due to their analysis of MIBC patients only, as well as the insurance-based nature of their healthcare system. 

A larger retrospective cohort (n = 29,010) found that social deprivation, as measured by the ADI, correlated strongly with a more advanced stage at presentation [9]. These findings are in line with other studies worldwide [19,20]. The ADI is very similar in structure to the SIMD. Both apply census data on social variables to create area rankings. Although SIMD stratifies areas into quintiles, the ADI utilises quartiles. Both tools were developed to better understand health disparities beyond individual patient factors. A more recent California-based cohort (n = 729) specifically evaluated the impact of socioeconomic status (as defined by a state-specific deprivation index) following radical cystectomy [12]. By virtue of their requiring a cystectomy, this cohort had more advanced disease on average than those in our cohort. However, they reported that patients in lower deprivation groups presented with more advanced disease.

This contrasts with our own data, which showed no significant difference in stage at presentation (NMIBC vs. MIBC) between SIMD groups. Reasons for this may centre around the lack of cost implications associated with universal healthcare systems. Countries with predominantly private healthcare systems may promote financial concerns among certain patient groups, particularly those in more deprived areas. Other studies support the notion that socioeconomically disadvantaged patients in the United States present with more advanced disease on average [9,12]. This may reflect apprehension over healthcare costs, translating into delays in diagnosis. However, a recent UK-based study reported findings that supported the concept of more deprived groups presenting with more advanced disease on average [21]. These findings were accrued in the context of a public health system. The reasons for this disparity with our cohort are unclear, and further research would be required to elucidate this.

The literature is conflicting regarding the prognostic differences between women and men. It is commonly reported that women have worse OS [11], for reasons that remain largely unexplained [15]. Andreassen et al. challenged this viewpoint. Their cohort study (n = 15,129) demonstrated that women have a worse prognosis exclusively within the first two years after diagnosis, particularly in the context of muscle-invasive disease. Thereafter, men are seen to have a worse prognosis [22]. To complement this, and with specific reference to deprivation, Jubber et al. reported a survival difference at three years of 8.9% in males and 13.4% in females between the top and bottom social deprivation quintiles, respectively [23].

It is well documented that females present with more advanced disease. This may partly be explained by delays in diagnosis due to similarities between bladder cancer and urinary tract infection symptoms [11]. In a large cohort study, Finnochiaro et al. recently reported that women were more likely to live in deprived areas and present with advanced disease [9]. Our data complements this, with a statistically significant increase in women presenting with muscle-invasive disease compared to men. However, in our cohort, this did not translate into a decrease in OS. The reasons for this are not clear, which is reflective of the conflicting nature of the existing literature. 

Comparison of our cohort study with others highlights discrepancies, underlining the need for more large-scale prospective studies. There is increasing use of multiple deprivation indices (MDI) to evaluate health outcomes in vulnerable populations. Significant variability exists in MDI methodology and the economic indicators assessed [13]. This reflects geographical and ethnic diversity across not just countries, but continents as well. 

The impact of social deprivation on health is multifaceted. It can be linked not just to worse health behaviours and higher rates of comorbidities, but also to healthcare access and the biological behaviours of cancers in different populations [24]. It is estimated that up to 40% of all cancers can be prevented through alterations in modifiable risk factors [25]. Smoking tobacco is the single biggest risk factor for developing bladder cancer. It has been shown that individuals living in more deprived areas are up to four times more likely to smoke than those living in more prosperous areas [26]. This impacts bladder cancer outcomes across multiple levels. Firstly, it is not surprising that other smoking-related diseases are more common in deprived areas. There is a higher incidence of cardiorespiratory disease that may render some patients unsuitable for more radical bladder cancer treatments.

According to the Office for National Statistics, an individual’s probability of smoking increases in line with their level of deprivation. Smokers’ cumulative exposure is an independent risk factor for both recurrence and progression of bladder cancer. An individual is more likely to smoke if they have no qualifications, are receiving benefits, or have health problems that limit their physical activity [27]. Again, this compounds further deprivation, increases comorbidity burden, and contributes to obesity from a sedentary lifestyle. There is also a strong link between obesity and cancer aetiology [25]. In Scotland, the prevalence of obesity has increased substantially in recent decades [28], with patients in the most deprived areas being almost twice as likely to be obese as those in less deprived areas [29]. Occupational exposure is also a well-recognised risk factor for bladder cancer, but is now less of an issue in developed nations following workplace legislation. 

Our study has several limitations. Firstly, this retrospective patient cohort is from a single region of Scotland, thus potentially limiting the generalisability of findings. A reliance on electronic health records may introduce misclassification errors. Possible confounding variables such as smoking intensity and occupational exposure were not captured in our database and so could not be analysed. We only correlated OS with SIMD and did not assess other endpoints such as CSS, recurrence-free survival (RFS), or comorbidity-adjusted outcomes. The latter of which can determine treatment eligibility and could be assessed by the Charleston Comorbidity Index. Patients were classified into NMIBC and MIBC groups for analysis. However, there does not appear to be any literature assessing socioeconomic status and the incidence of low, intermediate, and high-risk NMIBC. This could have formed the basis of a subgroup analysis. 

Although there is equity of treatment irrespective of socioeconomic status, these survival discrepancies cannot be adequately explained without a full complement of treatment data. Several studies, particularly from the United States, looked specifically at ethnicity, which is perhaps reflective of the heterogeneous nature of their respective populations. Ethnicity was not evaluated in our cohort, but this is felt unlikely to impact the data due to the ethnically homogenous nature of the Scottish population, which is 93% white based on recent census data [30]. There is also an ecological fallacy risk associated with studies that employ deprivation indices, meaning that not all individuals living in deprived areas are themselves deprived. The latter part of this study was conducted during the COVID-19 pandemic. It is difficult to extrapolate what impact local restrictions in bladder cancer care had on delayed presentations and outcomes. However, a UK-based study found that most patients did not experience a delay to their treatment or follow-up appointments [31]. 

There is a growing appreciation for the role of social determinants of health on cancer outcomes. More recent analyses are utilising robust, standardised census-based socioeconomic indicators to more accurately assess the influence of deprivation on cancer outcomes. Health inequalities need not be a default feature within our society. Addressing poverty and reducing socioeconomic disparity would not just improve survival outcomes from bladder cancer. Improvements in all health indices would be seen, with reduced downstream burden on the healthcare system. This study further highlights the need for public health interventions to support the delivery of equitable cancer care. Larger prospective trials are warranted to further clarify the link between social disparities and cancer outcomes.

## Conclusions

Within this cohort of bladder cancer patients, lower socioeconomic status as defined by the SIMD was associated with worse OS, conferring an approximately 25% higher hazard of death. These findings are in line with previous studies. Females were found to present with more advanced disease, which also aligns with existing literature. However, to the best of our knowledge, this is the first study to demonstrate that socioeconomic status in patients with bladder cancer does not appear to influence stage at presentation. This contradicts existing evidence and requires further investigation.

## References

[1] Marmot M, Bell R (2012). Fair society, healthy lives. Public Health.

[2] Gray AM (1982). Inequalities in health. The Black Report: a summary and comment. Int J Health Serv.

[3] (2025). Independent enquiry into inequalities in health report. https://www.gov.uk/government/publications/independent-inquiry-into-inequalities-in-health-report.

[4] (2025). The Dilnot Commission on social care. https://www.kingsfund.org.uk/insight-and-analysis/briefings/dilnot-commission-report-social-care.

[5] Hart JT (1971). The inverse care law. Lancet.

[6] Wilson S, Merville O, Dejardin O (2025). Use of mortality tables by level of deprivation in the study of social inequalities in cancer survival. Eur J Epidemiol.

[7] Leal J, Luengo-Fernandez R, Sullivan R, Witjes JA (2016). Economic burden of bladder cancer across the European Union. Eur Urol.

[8] Cox E, Saramago P, Kelly J (2020). Effect on bladder cancer on UK healthcare costs and patient health-related quality of life: evidence from the BOXIT trial. Clin Genitourin Cancer.

[9] Finocchiaro A, Tylecki A, Viganò S (2025). Socioeconomic disparities and bladder cancer stage at diagnosis: a statewide cohort analysis. JNCI Cancer Spectr.

[10] Shackley DC, Clarke NW (2005). Impact of socioeconomic status on bladder cancer outcome. Curr Opin Urol.

[11] Miller DT, Sun Z, Grajales V (2023). Insurance type and area deprivation are associated with worse overall mortality for patients with muscle-invasive bladder cancer. Urology.

[12] Dadabhoy A, Doshi C, Zahir M (2025). Impact of neighborhood deprivation on bladder cancer outcomes: a regional analysis. Bladder Cancer.

[13] Mogin G, Gorasso V, Idavain J (2025). A scoping review of multiple deprivation indices in Europe. Eur J Public Health.

[14] McCartney G, Hoggett R (2023). How well does the Scottish Index of Multiple Deprivation identify income and employment deprived individuals across the urban-rural spectrum and between local authorities?. Public Health.

[15] Scosyrev E, Trivedi D, Messing E (2010). Female bladder cancer: incidence, treatment, and outcome. Curr Opin Urol.

[16] Smith ND, Prasad SM, Patel AR (2016). Bladder cancer mortality in the United States: a geographic and temporal analysis of socioeconomic and environmental factors. J Urol.

[17] Boyd C, Zhang-Salomons JY, Groome PA, Mackillop WJ (1999). Associations between community income and cancer survival in Ontario, Canada, and the United States. J Clin Oncol.

[18] Bonner SN, Ibrahim AM, Kunnath N, Dimick JB, Nathan H (2023). Neighbourhood deprivation, hospital quality and mortality after cancer surgery. Ann Surg.

[19] Hasan S, Lazarev S, Garg M (2023). Racial inequity and other social disparities in the diagnosis and management of bladder cancer. Cancer Med.

[20] Rosenbaum CM, Filmar S, Gross AJ, Jobst N, Schultz A (2024). The influence of socioeconomic status and gender on incidence and survival in bladder cancer: a longitudinal study based on the Hamburg Cancer Registry. World J Urol.

[21] Barclay ME, Abel GA, Greenberg DC, Rous B, Lyratzopoulos G (2021). Socio-demographic variation in stage at diagnosis of breast, bladder, colon, endometrial, lung, melanoma, prostate, rectal, renal and ovarian cancer in England and its population impact. Br J Cancer.

[22] Andreassen BK, Grimsrud TK, Haug ES (2018). Bladder cancer survival: women better off in the long run. Eur J Cancer.

[23] Jubber I, Mitchell S, Hussain S, Tsoi H, Catto JW, Cumberbatch MG (2022). Social deprivation and bladder cancer: cause or effect for disparities in survival for affected women. BJU Int.

[24] Cumberbatch MG (2026). The role of social inequalities in the epidemiology of urological cancers: can this inform cancer screening?. Ann R Coll Surg Engl.

[25] Friedenreich CM, Ryder-Burbidge C, McNeil J (2021). Physical activity, obesity and sedentary behavior in cancer etiology: epidemiologic evidence and biologic mechanisms. Mol Oncol.

[26] (2026). Likelihood of smoking four times higher in England’s most deprived areas than least deprived. https://www.ons.gov.uk/peoplepopulationandcommunity/healthandsocialcare/drugusealcoholandsmoking/articles/likelihoodofsmokingfourtimeshigherinenglandsmostdeprivedareasthanleastdeprived/2018-03-14.

[27] (2026). Deprivation and the impact on smoking prevalence, England and Wales: 2017 to 2021. https://www.ons.gov.uk/peoplepopulationandcommunity/healthandsocialcare/drugusealcoholandsmoking/bulletins/deprivationandtheimpactonsmokingprevalenceenglandandwales/2017to2021#:~:text=A%20person's%20likelihood%20of%20smoking%20increased%20in,the%20level%20of%20deprivation%20in%20their%20neighbourhood..

[28] Tod E, Bromley C, Millard AD, Boyd A, Mackie P, McCartney G (2017). Obesity in Scotland: a persistent inequality. Int J Equity Health.

[29] (2026). The Scottish Health Survey 2022. https://www.gov.scot/publications/scottish-health-survey-2022-volume-1-main-report/pages/14/.

[30] (2026). The Scottish Public Health Observatory. https://www.scotpho.org.uk/population-groups/ethnic-minorities/data/population-composition/.

[31] Russell B, Spencer-Bowdage S, Rigby J (2022). The experience of UK patients with bladder cancer during the second wave of the COVID-19 pandemic. BJUI Compass.

